# TopLib: Building and Searching Top-Down Mass Spectral Libraries for Proteoform Identification

**DOI:** 10.1021/acs.analchem.4c06627

**Published:** 2025-05-29

**Authors:** Kun Li, Haixu Tang, Xiaowen Liu

**Affiliations:** 1 Deming Department of Medicine, 5783 Tulane University, New Orleans, Louisiana 70112, United States; 2 Luddy School of Informatics, Computing and Engineering, Indiana University, Bloomington, Indiana 47408, United States

## Abstract

Mass spectral library search is a widely used approach for spectral identification in mass spectrometry (MS)-based proteomics. While numerous methods exist for building and searching bottom-up mass spectral libraries, there is a lack of software tools for top-down mass spectral libraries. To fill the gap, we introduce TopLib, a new software package designed for building and searching top-down spectral libraries. TopLib utilizes an efficient spectral representation technique to reduce database size and improve query speed and performance. We systematically evaluated various spectral representation techniques and scoring functions for top-down spectral clustering and search. Our results demonstrate that TopLib is significantly faster and yields higher reproducibility in proteoform identification compared to conventional database search methods in top-down MS.

## Graphical abstract

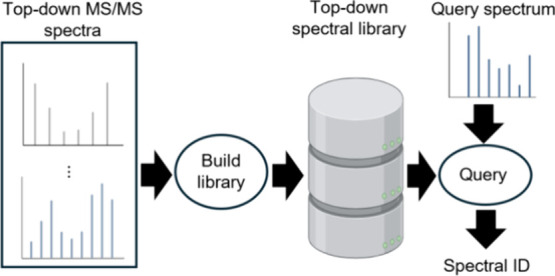

## Introduction

Mass spectrometry (MS) is a powerful technique for identifying and quantifying peptides, proteins, and proteoforms in complex biological samples.^1^ In a typical tandem mass spectrometry (MS/MS)-based proteomics experiment, two types of MS spectra are generated: MS1 spectra measure the molecular masses of peptides or proteoforms, and MS/MS spectra measure the mass-to-charge ratios (*m*/*z*) and intensities of fragments of peptides or proteoforms.^2^ There are two main approaches for identifying MS/MS spectra: database search and spectral library search.^3–6^ While database search methods compare query spectra against a protein/proteoform sequence database for spectral identification,^3,4^ spectral library search methods compare query spectra against the spectra of peptides or proteoforms collected in a prebuilt spectral library for spectral identification.^5,6^ Compared with database search, spectral library search leverages the intensity information on fragment ions observed in experimental spectra, enhancing the sensitivity in spectral identification.^7^

Bottom-up and top-down MS are two commonly used methods in MS-based proteomics.^8^ Bottom-up MS analyzes peptides generated through enzymatic digestion of proteins, while top-down MS directly examines intact proteoforms.^9^ In recent years, top-down MS has become the preferred method for identifying and characterizing intact proteoforms,^10^ but there is still a lack of software tools designed for building and searching top-down spectral libraries for proteoform identification. Although numerous approaches have been developed for building and searching spectral libraries in bottom-up MS,^6,11–13^ these methods cannot be directly applied to top-down MS due to the differences between the mass spectra generated in top-down and bottom-up MS. For example, top-down mass spectra typically contain more high charge state ions than bottom-up spectra, and spectral deconvolution,^14,15^ which converts a complex mass spectrum into a list of monoisotopic masses, is commonly required as a preprocessing step in top-down mass spectral analysis. Spectral library search has been incorporated into the software MetaMorpheus^16^ for bottom-up and top-down spectral identification, and the hybrid method in MetaMorpheus that combines database search and spectral library search slightly increased top-down spectral identifications.^17^

Efficient representation of mass spectra is critical for building and searching spectral libraries.^12^ While exploiting all fragment ions in MS/MS spectra enhances the sensitivity in spectral identification, it demands extensive storage space and slows down spectral library search. Efficiently representing mass spectral can greatly reduce the size of spectral libraries and accelerate spectral library search, without substantially compromising the sensitivity of spectral identification. Common approaches for representing spectra in bottom-up spectral libraries include bin-based methods,^11^ in which a mass spectrum is divided into *m*/*z* bins and each *m*/*z* bin is represented by its signal intensity, and deep learning models,^12^ which convert mass spectra into vector representations.

In spectral library-based methods, similarity scoring functions for ranking spectrum–spectrum–matches (SSMs) are essential for improving the accuracy of spectral clustering and the sensitivity of spectral identification. In bottom-up MS, various similarity or distance functions have been used for evaluating SSMs, including spectral dot product,^18,19^ Pearson correlation coefficient,^20^ Spearman’s rank correlation coefficient,^21^ and Euclidean distance.^12^ Additionally, a relative entropy-based distance function has been applied to spectral library searches in MS-based metabolomics.^22^

We introduce TopLib, a new software package designed for building and searching top-down spectral libraries. We conducted a systematic assessment of various spectral representation methods, similarity and distance functions, and spectral clustering algorithms for building and searching top-down spectral libraries. Experiments on top-down MS data generated from colorectal cancer cells demonstrated that spectral library search using TopLib identified many spectra missed by database search, significantly increased search speed, and enhanced the reproducibility of proteoform identification compared to database search.

## Methods

### Overview of TopLib

TopLib consists of four functions for building top-down mass spectral libraries (Figure 1). The first function is top-down spectral deconvolution^14,15^ (Figure 1a), which simplifies complex mass spectra by converting isotopic peaks of precursor and fragment ions into monoisotopic neutral masses. The second function identifies proteoform–spectrum–matches (PrSMs) by searching deconvoluted mass spectra against a protein sequence database^4,23,24^ (Figure 1b). TopFD^14^ and TopPIC^4^ are employed for spectral deconvolution and database search in TopLib. The third function builds a top-down comprehensive spectral library using deconvoluted top-down mass spectra, along with associated meta data and proteoform identification results stored in text files (Figure 1c). The fourth function groups mass spectra in the comprehensive library into clusters and generates a representative library containing one representative spectrum per cluster (Figure 1d).

**Figure 1:**
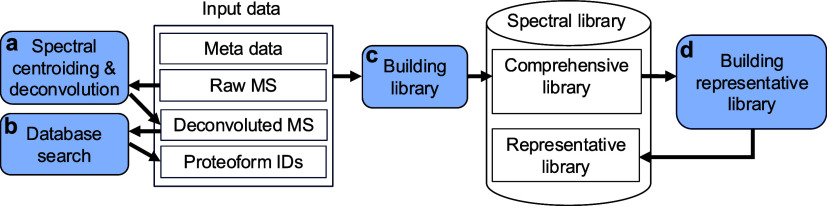
Overview of TopLib. Four functions are used for building top-down spectral libraries: (a) spectral centroiding and deconvolution, which convert mass spectra into monoisotopic neutral mass lists; (b) database searching of deconvoluted mass spectra for proteoform identification; (c) building a comprehensive library; and (d) generating a representative library from the comprehensive library. The representative library contains a representative spectrum for each cluster generated from the comprehensive library.

An SQLite database is used to store MS/MS spectra and proteoform identifications of top-down MS data in TopLib. The relational diagram depicting the tables of the database is given in Supplemental Figure S1. The precursor information and deconvoluted fragment masses of MS/MS spectra are stored in a spectrum and a mass table, respectively. Representative spectrum tables are used to store the information on representative spectra. Proteoforms matched to library spectra are stored in the ProForma format,^25^ and their UniProt accession numbers^26^ are stored in the database (Figure S1). TopLib also provides tools to convert them into libraries in the msalign text format^14^ and the standard NIST format.^27^

### Evaluation Data Sets

We generated two evaluation data sets using a top-down MS data set described in McCool et al.,^28^ in which three-dimensional (3D) separation coupled with top-down MS was utilized to analyze proteins extracted from SW480 cells. The data set included technical triplicates, and the first replicate, referred to as the SW480-3D data set, was used to generate the evaluation data sets.

In data preprocessing, the raw MS data files were converted to mzML files using msconvert (version 3.0.10765)^29^ and deconvoluted to msalign files using TopFD^14^ (version 1.7.5 and see Supplemental Table S1 for parameter settings). TopFD grouped precursor ions in each data file into proteoform features. The precursor ions in each proteoform feature had similar molecular masses and similar elution times in proteoform separation.

These msalign files reported by TopFD were subsequently searched against the UniProt human proteome sequence database (UP000005640_9606, 20,590 entries, version July 19, 2024) concatenated with a decoy database of the same size using TopPIC^4^ (version 1.7.5 and see Supplemental Table S2 for parameter settings). All proteoform–spectrum–matches (PrSMs) reported from the SW480-3D data set by database search were divided into proteoform groups using proteoform features reported by TopFD and proteoform identifications reported by TopPIC:^4^ Two PrSMs were assigned to the same proteoform group if the precursor ions of the two MS/MS spectra were from the same proteoform feature reported by TopFD or the two PrSMs were matched to the same protein and their precursor mass difference was no more than 10 ppm.

Because spectral deconvolution may introduce ±1 and ±2 Da errors into precursor masses of proteoforms, we removed possible duplicated proteoform groups as follows. All the proteoform groups were first ranked in the increasing order of the *E*-value based on their best PrSMs. For a proteoform group *A* matched to a protein *P*, if we found another proteoform group *B* such that (1) *B* was ranked higher than *A*, (2) *B* matched to protein *P*, and (3) the precursor mass difference between *A* and *B* was less than 2.2 Da, then the proteoform group *A* was removed.

We further removed PrSMs with inconsistent identifications. Two PrSMs were inconsistent if the two spectra were assigned to the same proteoform group, but they were matched to two different proteoforms. Note that these inconsistent PrSMs were assigned to the same proteoform group because their precursor ions were from the same proteoform feature reported by TopFD even though their proteoform identifications reported by database search were different. To remove inconsistent identifications, we ranked all PrSMs in the same proteoform group in the increasing order of the *E*-value. For a PrSM *A*, if we could find another PrSM *B* in the same proteoform group such that (1) *B* had a better *E*-value than *A* and (2) PrSMs *A* and *B* were matched to two different proteins, then PrSM *A* was removed.

The SW480-3D data set contained 54 MS data files and 75,605 MS/MS spectra, and a total of 37,566 PrSMs were identified with a 1% spectrum-level false discovery rate (FDR). After removing possible duplicated proteoform groups and inconsistent PrSMs, 28,913 PrSMs remained. The PrSMs were divided into 10,893 groups based on their proteoform identifications and charge states. The average size of the groups was 2.65 (Figure 2a). Then, we removed all groups with size 1, and the remaining 4359 groups with 22,379 spectra are referred to as the SW480-3D spectral groups.

**Figure 2:**
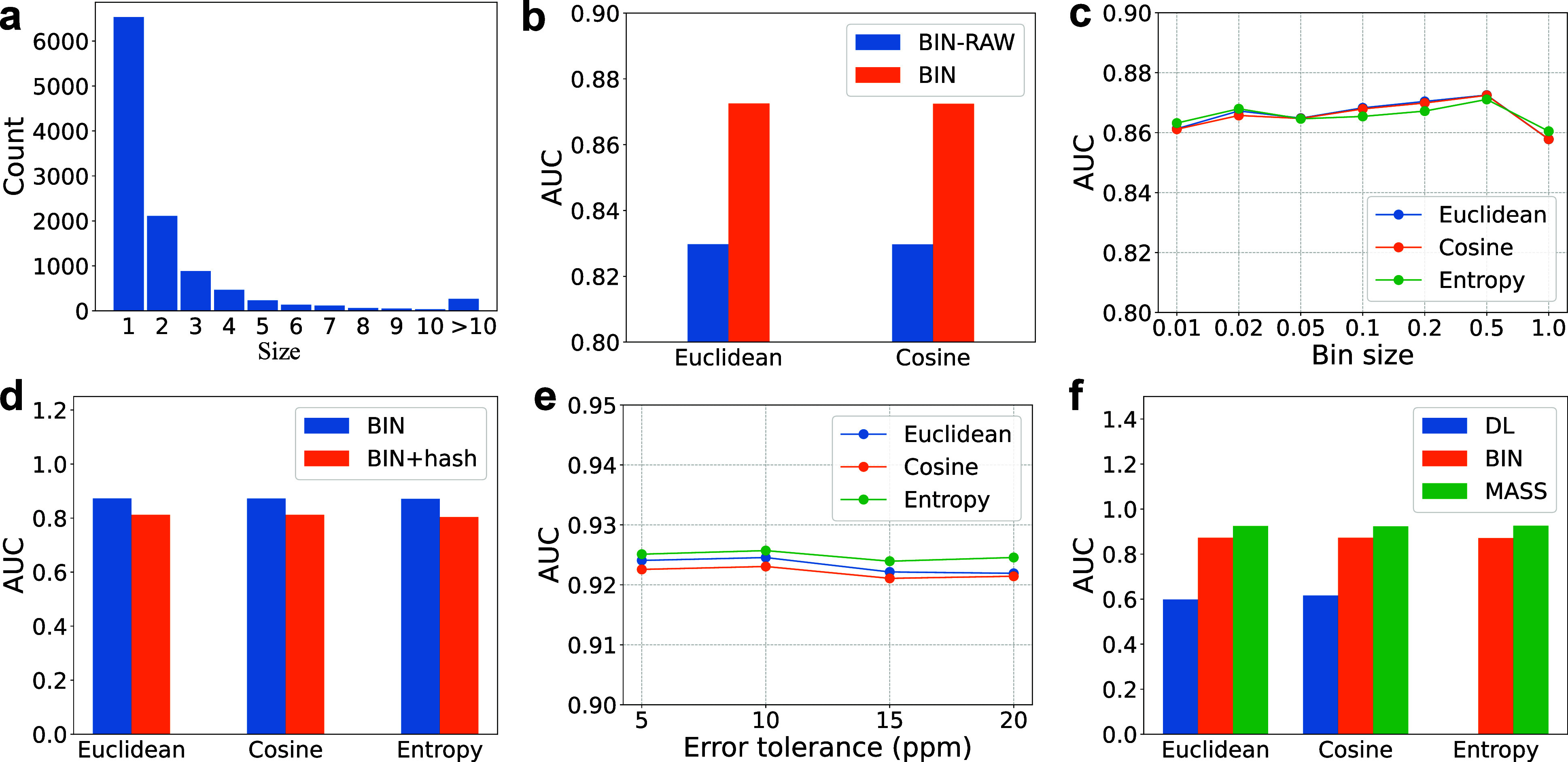
Evaluation of the DL, BIN, and MASS representation methods and Euclidean distance, cosine distance, and entropy-based distance for top-down mass spectra. The 50 highest-intensity deconvoluted fragment masses in each spectrum are used for spectral representation. (a) Distribution of the sizes of the 10,893 groups reported from the SW480-3D data set. (b) Comparison of the BIN-RAW and BIN representations on the SW480-SPE data set using a bin size of 0.5. (c) Comparison of various bin sizes in the BIN representation on the SW480-SPE data set. (d) Comparison of the BIN representation with and without a hashing function on the SW480-SPE data set using a bin size of 0.5. (e) Comparison of various settings for the error tolerance in the MASS representation on the SW480-SPE data set. (f) Comparison of the three spectral representation methods on the SW480-SPE data set with a bin size of 0.5 for the BIN representation and an error tolerance of 10 ppm for the MASS representation. The entropy-based distance is not used for the DL representation because mass intensity information is not available in the representation.

The first evaluation data set was generated to assess similarity and distance functions for SSMs. We randomly sampled 5000 spectrum pairs from same groups and 5000 spectrum pairs from different groups from the SW480-3D spectral groups. For each different-group spectrum pair, the precursor mass difference was restricted to the range of [0, 200] Dalton (Da). Cosine similarity, calculated using a bin-based spectral representation with a bin size of 0.5 (see Mass Spectral Representations), was employed to compare these two types of spectrum pairs. As expected, the cosine similarity scores were significantly higher for the same-group pairs compared with the different-group pairs (Supplemental Figure S2a). We further reduced the distinguishability between these two types of spectrum pairs by shifting some deconvoluted fragment masses in the same-group spectra to lower their similarity scores (Supplemental Figure S2b). Specifically, for each same-group spectral pair (*S*_1_, *S*_2_), spectrum *S*_1_ remained unchanged, and 90% of the fragment masses in *S*_2_ were shifted by random values within the ranges of [−200, −100] or [100, 200] Da. If a shifted mass was below zero or exceeded the precursor mass, a new random value within the ranges was selected to ensure that the shifted mass remained between 0 and the precursor mass. The evaluation set containing the 5000 same-group pairs with shifted masses and the 5000 different-group spectrum pairs is referred to as the SW480 spectral pair evaluation (SW480-SPE) data set.

The second evaluation set was designed to benchmark the performance of spectral clustering methods (see Top-Down Spectral Clustering). Since many SW480-3D spectral groups have distinct precursor masses, one group can easily be separated from others based solely on their precursor masses. To test the accuracy of spectral clustering when two or more groups share similar precursor masses, we changed the precursor masses of some spectra in the SW480-3D spectral groups, ensuring that most spectra could not be correctly clustered using precursor masses alone. For a given charge state *c*, we selected all SW480-3D spectral groups with the charge state *c.* For each spectral group, the spectrum with the best *E*-value PrSM was chosen as the representative spectrum. Then, we ranked the representative spectra using their precursor masses in the decreasing order. For *i* = 1, 3, 5, ···, we calculated the precursor mass difference between the *i*th and *i*+1th representative spectra and then increased the precursor masses of all spectra in the *i*+1th group by the mass difference. The resulting SW480-3D spectral groups with updated precursor masses are referred to as the SW480 group paired spectrum evaluation (SW480-GPSE) data set.

### Mass Spectral Representations

Since top-down MS/MS spectra often contained many isotopic peaks for each fragment ion, spectral deconvolution was employed to convert these isotopic peaks into neural monoisotopic masses to simplify the data. As a result, a deconvoluted mass spectrum can be viewed as a list of (mass, intensity) pairs or (*m*/*z*, intensity) pairs, where the *m*/*z* values correspond to the monoisotopic peaks of the deconvoluted masses. Three representation methods were employed to convert a deconvoluted mass spectrum into a vector of real numbers to speed up spectral similarity/distance computation. The first representation method is a deep learning-based encoding approach,^12^ where a deconvoluted spectrum with (*m*/*z*, intensity) pairs is converted into a vector of size 32. The second method allocates the intensities of deconvoluted masses to bins based on their *m*/*z* values.^11^ The third method simplifies each spectrum by retaining only the *k* most intense (mass, intensity) pairs for representation. These methods are referred to as DL (deep learning-based), BIN (bin-based), and MASS (mass intensity pair) representations.

For each spectrum, only the *k* most intense (mass, intensity) pairs were retained to simplify the data and the default setting for *k* was set to 50. In the DL representation, each mass in a spectrum is converted to its corresponding monoisotopic *m*/*z* value, the intensities are normalized to a unit length, and a deep neural network^12^ is utilized to encode the (*m*/*z*, intensity) pairs to a vector of size 32. In the BIN and MASS representations, log transformation (base 2) is applied to the intensities, and the log-transformed intensities are normalized to a unit length. In the BIN representation, masses are also converted to their corresponding monoisotopic *m*/*z* values, and a binning method, with a user-specified bin size, is utilized to convert the (*m*/*z*, log­(intensity)) pairs in the *m*/*z* range of [200, 1700] into a vector of log intensities,^11^ which is further normalized to a unit length. In the MASS representation, the spectrum is represented by its mass and normalized intensity pairs.

### Distance Functions for Mass Lists

In the MASS representation, two spectra *S* and *T* are represented as lists of (mass, intensity) pairs, in which the mass intensities in each spectrum are normalized to a unit length. An intensity *x* from *S* is matched to an intensity *y* is from *T* if the distance between their corresponding mass values is less than an error tolerance. Additionally, ±1.00235 Da errors are allowed in matching fragment masses. If an intensity in *S* or *T* cannot be matched to any intensity in the other spectrum, it will be paired with a zero intensity. Euclidean distance, cosine distance, and the entropy-based distance of the mass representations of *S* and *T* are defined on the two normalized unit vectors obtained from the paired intensities. To speed up the distance computation in TopLib, the mass intensities in *S* and *T* are normalized to a unit length before the paired intensities are found, and spectral distances are computed using these normalized intensities. Because one mass in one spectrum may be matched to multiple masses in the other, the normalized intensities computed based on paired intensities may be slightly different from those calculated based on single spectra.

### Top-Down Spectral Clustering

We developed TopCluster, a method for clustering top-down mass spectra. In TopCluster, mass spectra are clustered in three steps. (1) All spectra are grouped based on their precursor charge state, ensuring that the spectra in a group share the same-charge state. (2) The spectra in each group reported from step (1) are clustered based on their precursor masses: each spectrum is represented by only its precursor masses and clustered using hierarchical clustering with the complete linkage and a distance threshold of 2.2 Da. (3) In the final step, the spectral clustered reported from step (2) are further clustered using the fragment masses in their MS/MS spectra. Pairwise spectral cosine distances are calculated and then used as input for hierarchical clustering with average linkage or DBSCAN clustering.^30^

### Evaluation Metrics for Spectral Clustering

Clustering performance was evaluated using ARI,^31^ clustered spectra ratio, incorrect clustering ratio, and completeness.^11,12,32^ For two partitions *A* and *B* of the same set of spectra, ARI is computed as follows:

$$\mathrm{ARI}=\frac{2\left(ad-bc\right)}{\left(a+b\right)\left(b+d\right)+\left(a+c\right)\left(c+d\right)}$$ 1

where *a* is the number of spectral pairs placed in the same cluster by both partitions *A* and *B*; *b* is the number of pairs placed in the same cluster in partition *A*, but in different clusters in partition *B*; *c* is the number of pairs placed in the same cluster in partition *B* but in different clusters in partition *A*; and *d* is the number of pairs of placed in the different clusters by both partitions *A* and *B*.

A cluster with at least two spectra is called a valid cluster, and any spectrum assigned to a valid cluster is considered as a clustered spectrum. *The ratio of clustered spectra* of a partition of a set of spectra is the fraction of clustered spectra in the entire set.

Let *A* be the ground-truth partition of a spectral set and *C* is a valid cluster reported by a clustering method from the spectral set. We further divide *C* into subclusters based on the clusters in *A*: two spectra in *C* are assigned to the same subcluster if they are in the same cluster in *A*. The spectra in the largest subcluster are considered as correctly clustered ones; the remaining ones are incorrectly clustered. If multiple subclusters contain the same largest number of spectra, one is randomly selected as the “largest” one. *The ratio of incorrectly clustered spectra* of a partition is the ratio between the numbers of incorrectly clustered spectra and clustered spectra.

Consider a set of *N* spectra with a ground-truth partition *A* = {*A*_1_, *A*_2_, ···, *A*_*K*_} with *K* clusters and another partition *B =* {*B*_1_, *B*_2_, *···, B*_*L*_} with *L* clusters of. Let *n*_*j*_ be the total number of spectra in *B*_*j*_ and *n*_*i*,*j*_ the number of spectra shared by *B*_*i*_ and *A*_*j*_. The completeness of *B* with respect to *A* ^33^ is defined as $1-\frac{H\left(B|A\right)}{H\left(B\right)}$ , where

$$H\left(B|A\right)=-\sum_{j=1}^{L}\sum_{i=1}^{K}\frac{n_{i,j}}{N}\log\left(\frac{n_{i,j}}{n_{j}}\right)$$ 2

and

$$H\left(B\right)=-\sum_{j=1}^{L}\frac{n_{j}}{N}\cdot \log\left(\frac{n_{j}}{N}\right)$$ 3

### Building Spectral Libraries

We evaluated spectral library search-based proteoform identification using another top-down MS data set described in McCool et al.,^28^ in which two-dimensional (2D) size exclusion chromatography-capillary zone electrophoresis (SEC-CZE) separation coupled with top-down MS was employed to analyze proteins extracted from SW480 and SW620 cells. The data set included technical triplicates. The SW480 replicates are referred to as SW480-2D-1, SW480-2D-2, and SW480-2D-3, and the SW620 replicates are referred to as SW620-2D-1, SW620-2D-2, and SW620-2D-3.

To construct a top-down spectral library using MS data from a SW480 or SW620 replicate, raw MS files were preprocessed using msconvert^29^ and TopFD,^14^ and deconvoluted spectra were identified by database search using TopPIC^4^ using the same parameter settings in Evaluation Data Sets. MS/MS spectra lacking deconvoluted precursor information or containing fewer than two fragment masses were discarded. The remaining spectra were clustered using TopCluster (parameter settings in Supplemental Table S3).

Possible incorrect proteoform identifications reported by TopPIC were filtered following the method described in Evaluation Data Sets. Spectral clusters without any proteoform identifications reported by TopPIC were discarded. If the spectra in a cluster are matched several proteoforms, the proteoform with the best *E*-value PrSM reported by TopPIC was selected for the cluster. If two clusters were matched to the same proteoform with the same-charge state, only the cluster with the best *E*-value PrSM was retained, and the other was removed.

Two types of representative spectra were generated for a spectral cluster: single representative spectra and average representative spectra. Given a spectral cluster, if some spectra in the cluster had proteoform identifications reported by database search, the spectrum corresponding to the best *E*-value PrSM was selected as the single representative spectrum. Otherwise, the spectrum with the largest number of fragment masses was selected as the single representative spectrum.

To generate the average representative spectrum of a cluster, we first computed the merged spectrum *T* of two spectra in the cluster, and then the merged spectrum of *T* and the third spectrum. This process was repeated until the merged spectrum of all the spectra in the cluster was obtained. An error tolerance of 10 ppm was used to merge deconvoluted fragment masses in the spectra. For two masses *x*_1_ and *x*_2_ with their corresponding intensities *y*_1_ and *y*_2_, the merged mass was their weighted average mass *x* = (*x*_1_ *y*_1_ + *x*_2_ *y*_2_)/(*y*_1_+*y*_2_). Finally, the 50 most intense fragment masses in the merged spectrum were selected, their intensities were normalized to a unit length, and the resulting (mass, intensity) pairs were reported as the representative spectrum.

### Decoy Spectral Libraries

The target-decoy approach^34^ was used to estimate the FDRs of spectral identifications reported by spectral library search. For each representative spectrum in the spectral library, a decoy spectrum was generated using a mass shifting method. For each fragment mass in the representative spectrum, two different amino acids were randomly selected, and the fragment mass was shifted by the difference between their monoisotopic masses. If the shifted mass fell below zero or exceeds the precursor mass, a new random shift was considered until the shifted mass is between 0 to the precursor mass.

### Spectral Library Search of Escherichia coli MS Data

We searched mass spectra from an E. coli top-down MS data set^35^ against a spectral library built from the SW480-2D-1 data set to study incorrect spectral identifications reported by spectral library search (see Results). The E. coli top-down MS data set^35^ was downloaded from PRIDE (ID: PXD007273), which was acquired via CZE-MS/MS in duplicate. The raw files of the two replicates were preprocessed using the same methods described in Evaluation Data Sets. The deconvoluted spectra of the two replicates were then merged and searched against the spectral library using the following parameter settings: a precursor mass error tolerance of 100 Da, no precursor charge matching was required, and no cosine similarity-based filtering was applied.

## Results

### Spectral Representations and Distance Functions

We evaluated three spectrum representation methods (see Methods), referred to as DL (deep learning-based), BIN (bin-based), and MASS (mass intensity pair), for deconvoluted top-down MS/MS spectra. We first used the SW480-SPE data set to compare the performance of the BIN representation (with log transformation) and the bin-based representation without log transformation of mass intensities (see Methods), referred to as BIN-RAW. For a spectral pair with a cosine similarity *s*, the cosine distance is defined as 1 – *s*. Pairwise spectral distances were computed using Euclidean distance, cosine distance, or an entropy-based score^22^ (see Methods). Compared with BIN-RAW, BIN demonstrated better discriminative power in distinguishing between same-group and different-group spectral pairs using Euclidean and cosine distances (Figure 2b). Consequently, log transformation of mass intensities was used as the default setting for computing Euclidean and cosine distances for the BIN and MASS representations. However, it was not applied to the entropy-based score, as log transformation is already integrated into the score. For the DL representation, we used the method proposed by Bittremieux et al.^12^ without applying intensity log transformation.

We then evaluated the BIN representation with various settings for the bin size on the SW480-SPE data set. The highest area under receiver operating characteristic curve (AUC) value was achieved with a bin size of 0.5 Thomson (Th) for Euclidean distance, cosine distance, and the entropy-based score (Figure 2c). Based on the results, the bin size 0.5 was selected as the default setting for the BIN representation. Additionally, we experimented a hash function^11^ to reduce the BIN representation (the vector size is 3000 for the default *m*/*z* range [200, 1700] Th with a bin size of 0.5) to a small 800-dimensional vector. However, the hash function significantly reduced the discriminative power compared with the representation without hashing (Figure 2d).

We also assessed various error tolerance settings for matching deconvoluted fragment masses in the MASS representation on the SW480-SPE data set. The distribution of the errors of fragment masses in the 5000 same-group spectrum pairs (Supplemental Figure S3) indicated that ±1 Da errors are common in deconvoluted fragment masses. Therefore, ±1 Da errors were allowed in matching fragment masses. In addition, only masses with the same-charge state were matched. That is, two mass charge pairs (*m*_1_, *c*_1_) and (*m*_2_, *c*_2_) were considered to be matched if (1) *c*_1_ = *c*_2_ and (2) |*m*_1_ – *m*_2_| < *e* or 1.00235 – *e* < |*m*_1_ – *m*_2_| < 1.00235 + *e*, where 1.00235 Da is an estimated average mass difference between two neighboring isotopic masses of a fragment.^36^ The best AUC was obtained with an error tolerance of 10 ppm (Figure 2e), which was chosen as the default error tolerance for the MASS representation.

We further evaluated the discriminative ability of the three representation methods using the default error tolerance and various settings for the number *k* of fragment masses kept in a mass spectrum: 25, 50, 75, 100, 150, and 200 (see Methods) on the SW480-SPE data set. The highest AUC was obtained at *k* = 50 for the BIN and DL representations and at *k* = 100 for the MASS representation (Supplemental Tables S4–S6). As a result, we selected *k* = 50 as the default setting for both the BIN and DL representations. Although *k* = 100 yielded the best AUC for the MASS representation, we opted to use *k* = 50 as the default setting to reduce the spectral library size. Furthermore, experimental results for spectral clustering (see Spectral Clustering) indicated that *k* = 50 provided better clustering performance than *k* = 100.

Using the default settings, the MASS representation achieved the highest discriminative ability among the three methods (Figure 2f), indicating that the DL and BIN representations may lose some information on the deconvoluted masses compared with the MASS representation, reducing their ability to accurately differentiate the same-group and the different-group spectrum pairs. For the MASS representation, no significant differences were observed among the three distance metrics.

### Spectral Clustering

TopCluster was evaluated using the three spectral representation methods with their default parameter settings and benchmarked against spectra-cluster (version 1.1.2)^37^ on the SW480-GPSE data set with 22,379 spectra from 4359 clusters. In the spectra cluster, a probabilistic score serves as the similarity function for spectral pairs, and a greedy approach is employed for clustering.

The clusters in the SW480-GPSE data set, generated based on proteoform identifications reported by TopPIC^4^ (see Methods), were used as the ground truth in the evaluation. Clustering performance was assessed using three metrics: the ratio of clustered spectra, the ratio of incorrectly clustered spectra, and the completeness of clustering (see Methods). TopCluster with the BIN and MASS representations and hierarchical clustering outperformed other methods by producing a higher ratio of clustered spectra and achieving better completeness for a given ratio of incorrectly clustered spectra (Figure 3a,b). TopCluster also demonstrated comparable performance with Euclidean distance and the entropy-based score (Supplemental Figure S4) and with the DBSCAN clustering method (Figure 3d,e and Supplemental Figure S5). Additionally, we compared the performance of the MASS representation with two settings for the parameter *k* (50 and 100) and found that *k* = 50 outperformed *k* = 100 in clustering accuracy (Supplemental Figure S6).

**Figure 3:**
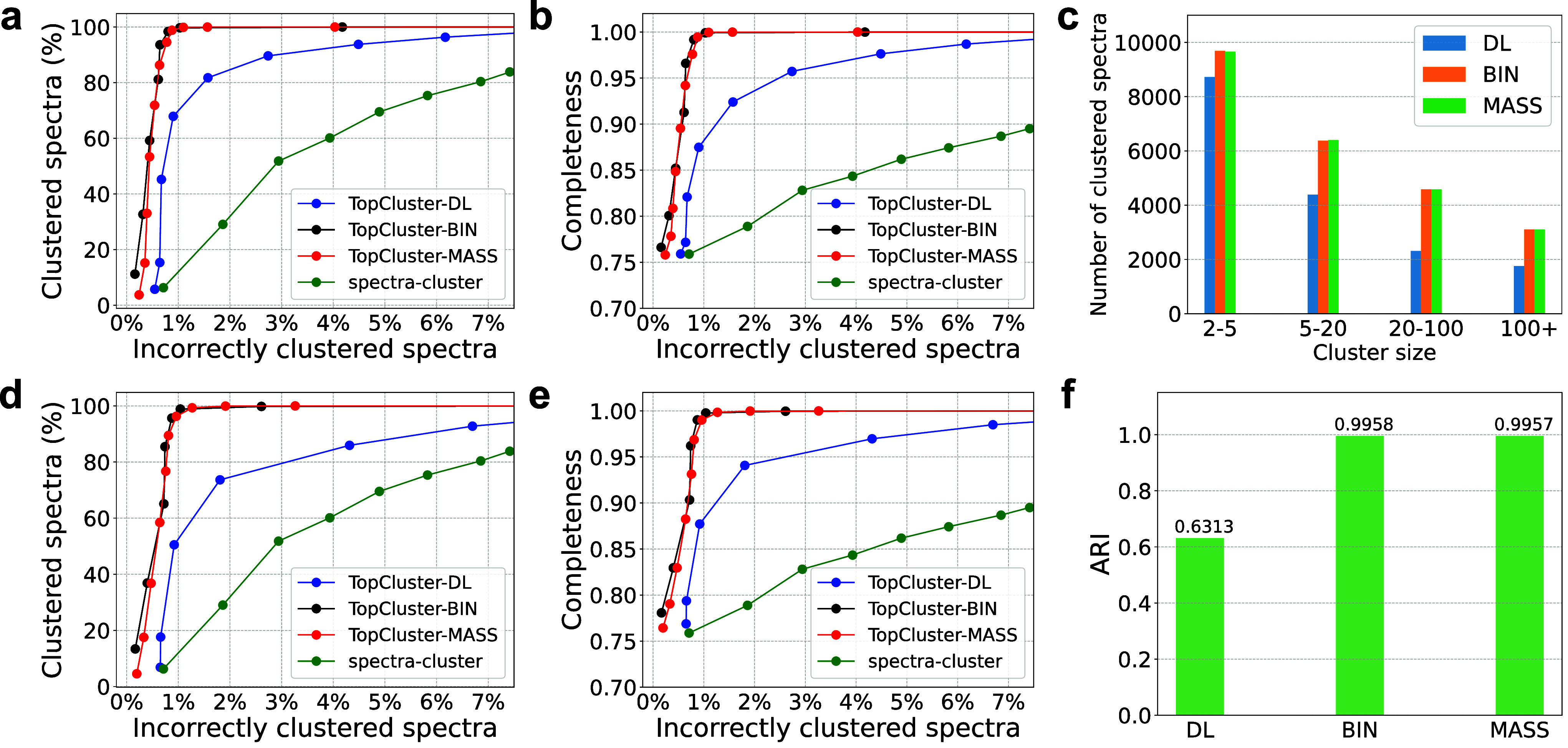
Comparison of spectral clustering accuracy of spectra-cluster and TopCluster using three spectral representation methods on the SW480-GPSE data set. (a) and (d) show the ratio of incorrectly clustered spectra against the ratio of clustered spectra for hierarchical clustering and DBSCAN, respectively. (b) and (e) plot the ratio of incorrectly clustered spectra against clustering completeness for hierarchical clustering and DBSCAN, respectively. (c) and (f) give the sizes and ARIs of the clusters reported by TopCluster using distance cutoffs of 0.161 for the DL representation, 0.796 for the BIN representation, and 0.876 for the MASS representation.

We further compared the performance of the three representation methods using a cosine distance cutoff of 0.161 for the DL representation, 0.796 for the BIN representation, and 0.876 for the MASS representation. At these cutoffs, the three methods reported similar ratios of incorrectly clustered spectra: 1.00% for DL, 1.02% for BIN, and 1.01% for MASS. TopCluster with the BIN and MASS representations identified more nonsingleton clusters (BIN: 4265, MASS: 4267) compared with the DL representation (3796) (Figure 3c). Additionally, the nonsingleton clusters reported by the BIN and MASS representations contained more spectra (BIN: 22,307; 99.68%, MASS: 22,325; 99.76%) than those from the DL representation (16,042; 71.68%), and the adjusted Rand index (ARI)^31^ of the clusters reported by the MASS and BIN representations was also higher than that reported by the DL representation (Figure 3f).

### Proteoform Identification by Spectral Library Search

A top-down spectral library was built using the SW480-2D-1 data set with 22,455 MS/MS spectra (see Methods). TopCluster (parameter settings in Supplemental Table S3) was employed to group these spectra into 13,016 spectral clusters. These clusters were further filtered based on proteoform identifications reported by database search using TopPIC,^4^ reducing the total number of clusters to 5155. A representative spectrum was computed for each of the 5155 clusters by averaging the deconvoluted spectra in each cluster (see Methods). The final set of 5155 representative spectra, corresponding to 3773 proteoforms, are referred to as the SW480-2D-1 library. To estimate the FDR of identifications, a decoy SW480-2D-1 library of the same size was also built (see Methods).

We searched the 22,924 MS/MS spectra in SW480-2D-2 against the SW480-2D-1 spectral library using four combinations of precursor matching parameters: precursor mass error tolerance (10 ppm or 2.2 Da) and whether precursor charge matching was applied. Each query spectrum was searched against the spectral library to find the representative spectrum with a matched precursor mass and the highest cosine similarity score. Spectral identifications reported from the library search were filtered using a cosine similarity cutoff of 0.3. Increasing the precursor mass error tolerance and permitting matches between precursors with different-charge states resulted in more spectral identifications (Supplemental Figure S7).

We evaluated the error rates of spectral library search results based on inconsistent spectral identifications reported by spectral library search and database search. The identifications of a spectrum are inconsistent if the spectrum was matched to two different proteins by the two methods. The error rate of spectral identifications reported by spectral library search was estimated as the ratio of the number of inconsistent identifications to the number of identifications shared by the two search methods, referred to as the database search inconsistency (DSI) error rate. DSI errors may arise from false proteoform annotations in the spectral library, inaccuracies in the SW480-2D-2 database search results, or errors in the spectral library search itself, so the actual error rate for spectral library search is lower than the DSI error rate.

Removing the precursor charge matching requirement increased the number of spectral identifications without significantly affecting the estimated DSI error rates. Increasing the precursor error tolerance resulted in more identifications but also higher estimated error rates (Figure 4a and Supplemental Figure S7). Based on these findings, we selected the default precursor matching parameters as follows: a 10 ppm precursor error tolerance and no requirement for precursor charge matching.

**Figure 4:**
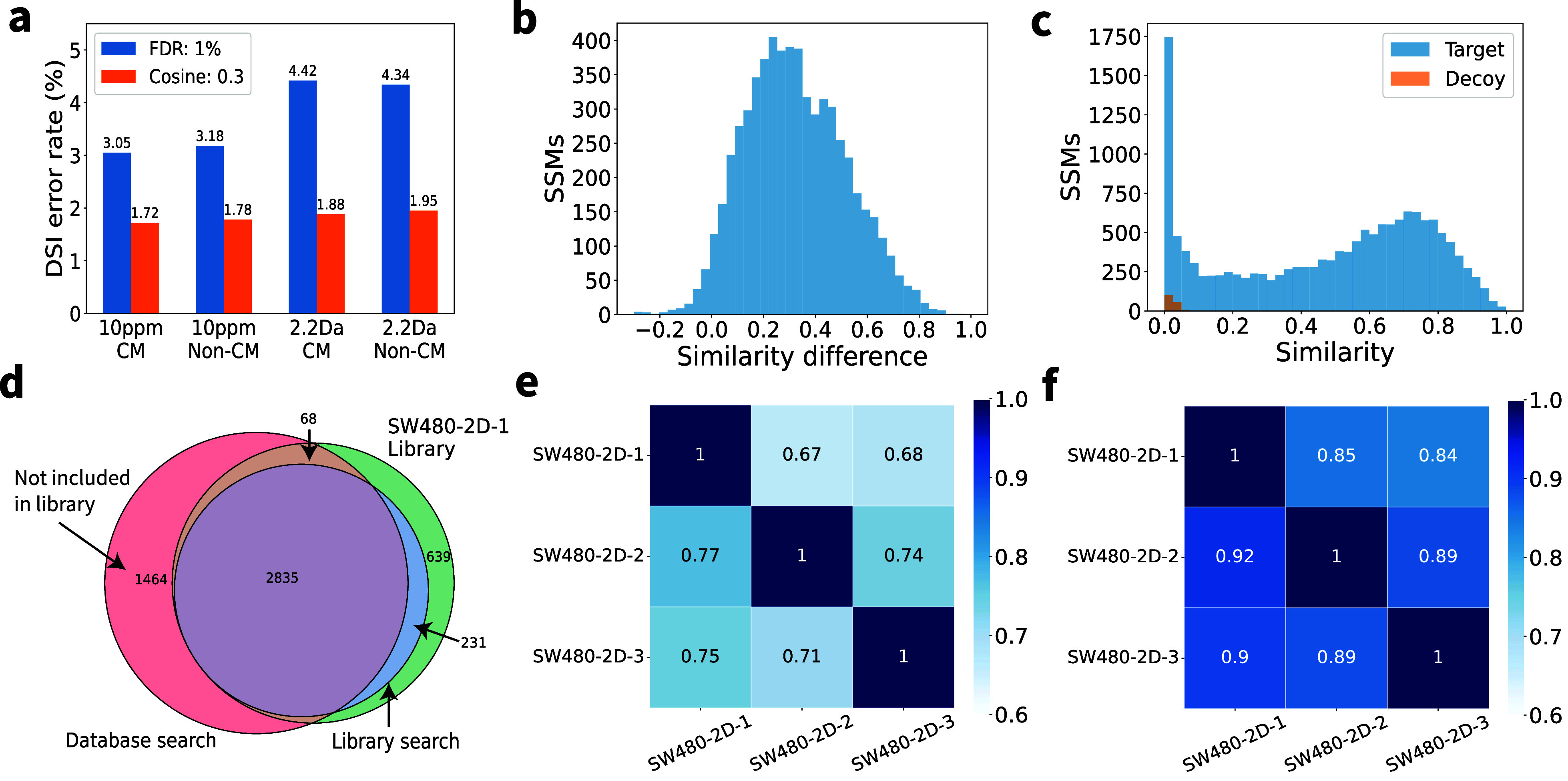
Evaluation of spectral library search. (a) DSI error rates of spectral library search results reported by searching the spectra in SW480-2D-2 against the SW480-2D-1 library with four parameter combinations: a precursor mass error tolerance of 10 ppm or 2.2 Da, and with or without precursor charge matching (CM or Non-CM). Two quality control methods are used: 1% spectrum-level FDR or a cosine similarity cutoff of 0.3 (cosine: 0.3). (b) Distribution of cosine similarity score differences between SC-SSMs and DC-SSMs for the 6066 query spectra. (c) Distributions of cosine similarity scores for target and decoy SSMs identified by searching spectra from SW480-2D-2 against the target-decoy SW480-2D-1 spectral library. (d) Comparison of proteoforms in the SW480-2D-1 library, proteoform identifications reported from SW480-2D-2 by database search, and those by spectral library search. (e) Reproducibility of proteoform identifications in the SW480-2D triplicates using database search. (f) Reproducibility of proteoform identifications in the SW480-2D triplicates using spectral library search.

We further compared the similarity scores of SSMs with the same precursor charge state and those with different precursor charge states, referred to as same-charge SSMs (SC-SSMs) and different-charge SSMs (DC-SSMs), respectively. We searched the spectra in SW480-2D-2 against the SW480-2D-1 library with a precursor mass error tolerance of 10 ppm, precursor charge matching, and a cosine similarity cutoff of 0.3. For each identified SC-SSM, we then searched the SW480-2D-1 library for spectra from the same proteoform but with a different precursor charge state. The query spectrum in the SC-SSM and its matched library spectrum with a different precursor charge state were reported as a DC-SSM. If multiple DC-SSMs were reported for an SC-SSM, only one was randomly selected and reported. Finally, we obtained an evaluation set of 6066 query spectra from the SW480-2D-2 data set, each of which had both an SC-SSM and a DC-SSM in the SW480-2D-1 spectral library. The average cosine similarity scores of the SC-SSMs and DC-SSMs were 0.67 and 0.34, respectively (Supplemental Figure S8). For each of the 6066 spectra, we also computed the difference between the cosine similarity scores of its SC-SSM and DC-SSM. The average difference was 0.33 (Figure 4b), indicating that the similarity score of a DC-SSM may be substantially lower than that of an SC-SSM.

### Quality Control of Spectral Identifications

We used the target-decoy approach (see Methods) to determine a cosine similarity cutoff for filtering identifications reported by spectral library search. The 22,924 MS/MS spectra in SW480-2D-2 were searched against the SW480-2D-1 spectral library combined with the decoy SW480-2D-1 spectral library using default precursor matching parameters, and all spectral identifications were reported without filtering. In total, 15,273 target SSMs and 168 decoy SSMs were identified. The distributions of cosine similarity scores for the target and decoy SSMs indicated that a cutoff of 0.3 effectively separated target from decoy identifications (Figure 4c). Visual inspection of SSMs with a cosine similarity score of 0.3 revealed that the number of matched fragment masses in these SSMs was within an acceptable range (Supplemental Figure S9). When the default precursor matching parameter settings (10 ppm, precursor charge state matching not required) were used and a cosine similarity cutoff of 0.3 was applied, the DSI error rate was below 2% (Figure 4a). In contrast, with a 1% spectrum-level FDR, the corresponding cosine similarity cutoff was only 0.02, and the DSI error rate exceeded 3.18%, suggesting that FDRs estimated using the target-decoy approach may be underestimated (Figure 4a).

We also investigated the cosine similarity scores of incorrect SSMs by searching an E. coli top-down MS data set^35^ containing 9830 MS/MS spectra against the SW480-2D-1 spectral library (see Methods). Both the SW480 and E. coli data sets were acquired using Thermo Q Exactive mass spectrometers. A total of 4769 SSMs were reported, and the distribution of their cosine similarity scores showed that 4768 (99.9%) had a similarity score below 0.3, while only one SSM had a score of 0.32, exceeding the 0.3 threshold (Supplemental Figure S10). Based on these results, a cutoff of 0.3 was selected as the default cosine similarity threshold.

### Representative Spectra

We also compared two approaches for generating representative spectra for spectral clusters in the SW480-2D-1 spectral library: average and single representative spectra (Methods). Using average representative spectra reported more spectral identifications from SW480-2D-2 by spectral library search compared with single representative spectra (Supplemental Figure S11), suggesting that average spectra provide better representative spectra for spectral clusters than single spectra. As a result, average representative spectra were selected as the default in TopLib.

### Multiplexed Spectra

We examined the 5155 representative spectra in the SW480-2D-1 spectral library to identify possible multiplexed (chimeric) MS/MS spectra generated from coisolated precursor ions. For each spectrum, we calculated the total intensity of all isotopic peaks within the isolation window for each precursor, referred to as the isolation window intensity (ISI), and used the ISI to rank all precursors in the window. The ratio of the ISI of the top-ranked precursor to the total ISI of all precursors was defined as the primary precursor intensity ratio (PPIR) of the spectrum. The PPIR exceeded 50% in more than 90% of the spectra and exceeded 80% in over 62% of the spectra (Supplemental Figure S12a,b). We further searched the 5155 spectra against the UniProt human proteome database using TopMPI,^38^ a tool capable of identifying two proteoforms from a multiplexed spectrum. Of the 5155 spectra, TopMPI mapped 408 (7.9%) to proteoform pairs instead of single proteoforms, indicating that these 408 spectra are likely multiplexed.

To address the issue of multiplexed spectra, we applied a PPIR cutoff to exclude potentially multiplexed spectra. Spectral libraries were generated from the SW480-2D-1 data set using different PPIR cutoff values, and spectra from SW480-2D-2 were then searched against these libraries for spectral identifications. The results showed that increasing the PPIR cutoff reduced the number of spectral identifications, as more multiplexed spectra were excluded during library construction (Supplemental Figure S12c). In practice, the PPIR cutoff needs to be selected to balance the completeness and quality of the resulting spectral libraries.

### Evaluation of Spectral Deconvolution Methods

We compared the performance of TopLib coupled with TopFD^14^ (version 1.7.5, parameter settings in Supplemental Table S1) and FLASHDeconv^15^ (version 2.0, parameter settings in Supplemental Table S7), two spectral deconvolution tools, for building and searching top-down spectral libraries. Replacing TopFD with FLASHDeconv, we constructed a spectral library from the SW480-2D-1 data set and searched spectra from SW480-2D-2 against the library using default parameter settings. TopLib with FLASHDeconv identified a slightly smaller number of spectra compared to TopLib with TopFD (Supplemental Figure S13). We further evaluated two hybrid spectral library search workflows by building a library from SW480-2D-1 using FLASHDeconv and deconvoluting query spectra in SW480-2D-2 with TopFD, and *vice versa*. These hybrid approaches yielded slightly fewer spectral identifications than using TopFD or FLASHDeconv alone (Supplemental Figure S13).

### Comparison between Database Search and Spectral Library Search

We compared spectrum and proteoform identifications reported from SW480-2D-2 by database search using TopPIC (version 1.7.5, parameter settings in Supplemental Table S2) and by spectral library search using TopLib with the default parameter settings. TopLib identified 231 proteoforms and 1128 spectra missed by database search (Figure 4d and Supplemental Figure S14). This improvement is attributed to the inclusion of mass intensity information in the library spectra, which enhances the sensitivity of spectral library search in comparison with database search. On the other hand, TopLib missed 2738 spectra and 1532 proteoforms that were identified by database search. The primary reason for these missed identifications was the incompleteness of the spectral library: 1464 (95.6%) of the 1532 missed proteoforms were due to missing library spectra. Of the remaining 68 missed identifications, 48 were due to large errors in precursor masses, and 20 were due to low MS/MS spectral similarity.

TopLib was 140 times faster than TopPIC for spectral identification (TopLib: 3.35 min vs TopPIC: 470 min) (Supplemental Figure S15). We also replaced TopPIC with MSPathFinder^23^ for identifying spectra in the SW480-2D-2 data set by database search (parameter settings in Supplemental Table S8), and TopLib was 46.6 times faster than MSPathFinder (156 min). Additionally, the running time of TopFD for spectral deconvolution was approximately 75 min, and the total running time of the TopFD+TopLib pipeline (78 min) was about seven times faster than the TopFD+TopPIC pipeline (545 min) and 2.9 times faster than the TopFD+MSPathFinder pipeline (231 min) (Supplemental Figure S15).

Next, we evaluated the reproducibility of proteoform identifications reported by database search using TopPIC and by spectral library search using TopLib across six SEC-CZE data sets (three from SW480 cells and three from SW620 cells) described by McCool et al.^28^ The first two data sets, referred to as SW480-2D-1 and SW480-2D-2, were used in the previous section, while the other four are referred to as SW480-2D-3, SW620-2D-1, SW620-2D-2, and SW620-2D-3. We searched the MS/MS spectra in the six data sets against the UniProt human proteome database to identify spectra and proteoforms using TopPIC. For spectral library search, we built a spectral library using the SW620-2D-1 data set (see Methods) and searched the MS/MS spectra in SW480-2D-1, SW480-2D-2, and SW480-2D-3 against the spectral library separately to identify spectra and proteoforms using TopLib with the default parameter settings. Similarly, the MS/MS spectra in SW620-2D-1, SW620-2D-2, and SW620-2D-3 were searched against a spectral library built using the SW480-2D-1 data set for spectral identification. For each data set pair *A* and *B* in the SW480 or SW620 triplicates, the reproducibility of proteoform identifications for database or library search was computed as the ratio of the number of identifications shared by the two data sets to the number of identifications reported from *A*. On average, spectral library search improved the reproducibility of proteoform identifications by 15.6% compared to database search (Figure 4e,f and Supplemental Figure S16).

We also compared the performance of TopLib and TopPIC on a top-down MS data set generated using a Bruker maXis II (TOF) mass spectrometer (PRIDE ID: PXD019368).^39^ In the MS experiments, human embryonic kidney (HEK-293T) cells were analyzed with two membrane protein extraction methods separately: Tergitol NP-7 and Triton X-114. Using TopLib, we built a spectral library from the Tergitol data set and searched the 16,143 spectra from the Triton data set against the Tergitol spectral library for spectral identification with the default parameter settings. The resulting spectral library contained 217 representative spectra and a total of 719 spectra were identified from the Triton data set (Supplemental Figure S17). We also searched the Triton MS data against the UniProt human proteome database (version July 19, 2024; 20,590 entries) for spectral identification using TopPIC. Both database search and library search reported a low identification rate for the query spectra. TopLib identified 125 spectra that were missed by the database search and failed to identify 1068 spectra and 60 proteoforms that were identified by the database search. Among these 60 proteoforms, 46 were missed due to the incompleteness of the spectral library (Supplemental Figure S17). Of the remaining 14 missed identifications, 3 were attributed to large precursor mass errors, and 11 were due to low MS/MS spectral similarity between the library and query spectra.

## Discussion

We developed TopLib, a software tool for building and searching top-down spectral libraries for proteoform identification. TopLib leverages fragment mass signal intensities in top-down MS/MS spectra to enhance the sensitivity of spectral identification. As a result, TopLib improves the reproducibility of proteoform identifications compared with database search-based methods. Additionally, TopLib is substantially faster than TopPIC^4^ and other database search tools for top-down mass spectral identification.

TopLib uses deconvoluted top-down MS/MS spectra instead of nondeconvoluted ones to build and search spectral libraries. The main reason is that a fragment in nondeconvoluted spectra tends to have many isotopic peaks, making spectral library search inefficient. In top-down MS, it is common for a fragment to have more than five isotopic peaks, and a nondeconvoluted mass spectrum often contains more than 1000 isotopic peaks. For example, the average number of peaks in the nondeconvoluted spectra in the SW480-2D-1 library (5155 spectra; see Results) is 1324 (Supplemental Figure S18). One main disadvantage of deconvoluted masses is that the deconvolution process often introduces ±1 Da errors into deconvoluted fragment masses. To address this problem, ±1 Da errors can be allowed in fragment mass matching during spectral library search (see Results).

We compared the performance of three spectral representation methods, DP, BIN, and MASS, and found that the MASS representation outperformed the other two in distinguishing between SSMs from the same proteoform and those from different proteoforms. Additionally, applying a log transformation to mass signal intensities enhanced the ability to distinguish between these two types of SSMs. We demonstrated that spectra containing only the 50 most intense fragment masses achieved performance comparable to spectra with all deconvoluted fragment masses for spectral identification using spectral library search. The MASS representation also resulted in higher accuracy in spectral clustering than the other representation methods. Furthermore, for the MASS representation, no significant differences were observed across Euclidean distance, cosine distance, and the entropy-based distance.

We also comprehensively evaluated the performance of TopLib for spectral identification with various parameter settings. Using inconsistent identifications reported by database search and TopLib, we found that the FDR estimation based on target and decoy spectra may underestimate the error rate of the identifications reported by spectral library search. To address this issue, further research is needed to explore alternative methods for generating decoy spectra and estimating FDRs. In addition, removing the requirement for precursor charge state matching can increase spectral identifications without significantly increasing the error rate.

TopLib still has several limitations. First, it relies on comprehensive spectral libraries for spectral identification, restricting its applications in discovery-mode studies. Second, TopLib currently does not support querying spectra with unexpected mass shifts. Third, library spectra in TopLib are not fully annotated. TopLib uses PrSMs reported by database search to build spectral libraries, and localizing PTMs in database search remains a challenging problem due to low proteoform sequence coverage of fragment ions in top-down MS/MS spectra. As a result, we still lack an annotated spectral library with confident PTM localization, which makes it difficult to assess whether TopLib can confidently localize PTMs when the library contains several proteoforms of the same protein with the same PTM but different PTM sites. To address these challenges, manual inspection and new proteoform characterization methods are needed to enhance spectral annotation in these libraries.

## Supplementary Material



## Data Availability

The MS raw data can be downloaded from the PRIDE repository with the data set identifiers PXD029703 and PXD019368. The source code of TopLib is available at https://github.com/toppic-suite/toplib.

## Notes

(Figure S1) Database scheme diagram for storing MS data in TopLib; (Figure S2) distributions of cosine similarity of same-group and different-group spectrum pairs in the SW480-SPE data set; (Figure S3) distribution of the errors of matched fragment masses in the same-group spectrum pairs in the SW480-SPE data set; (Figure S4) comparison of spectral clustering accuracy of TopCluster with the BIN representation and hierarchical clustering using three distance functions: Euclidean, Cosine, and the entropy-based distances; (Figure S5) comparison of spectral clustering accuracy between hierarchical clustering and DBSCAN using TopCluster with the MASS representation; (Figure S6) comparison of spectral clustering performance between *k* = 50 and *k* = 100 for TopCluster using the MASS representation; (Figure S7) comparison of identifications reported from spectral library searches with four parameter settings for precursor matching; (Figure S8) distributions of the cosine similarity scores of the SC-SSMs and DC-SSMs of the 6066 query spectra; (Figure S9) examples of SSMs with a similarity score of 0.3; (Figure S10) distribution of cosine similarity scores for the 4769 SSMs reported by searching the E. coli data set against the SW480-2D-1 spectral library; (Figure S11) comparison of single representative and average representative spectra for spectral identification by searching the mass spectra in SW480-2D-2 against the SW480-2D-1 library; (Figure S12) PPIRs of the 5155 spectra in the SW480-2D-1 library; (Figure S13) comparison of spectral identifications reported from spectral library searches using different spectral deconvolution tools: TopFD and FLASHDeconv; (Figure S14) comparison of spectral identifications reported from SW480-2D-2 using spectral library search against the SW480-2D-1 library and database search; (Figure S15) comparison of the running times for TopLib, MSPathFinder, and TopPIC in identifying spectra in the SW480-2D-2 data set; (Figure S16) comparison of the reproducibility of proteoform identifications reported by database search and spectral library search; (Figure S17) comparison of database and library search results from the Triton data set; (Figure S18) distribution of the number of peaks in the 5155 nondeconvoluted MS spectra from the SW480-2D-1 library; (Tables S1–S3) parameter settings for TopFD, TopPIC, and TopCluster; (Tables S4–S6) comparison of different settings of *k* using the BIN, DL, and MASS representations on the SW480-SPE data set; and (Tables S7 and S8) parameter settings for FLASHDeconv and MSPathFinder (PDF)
