# Supplementary material for: TopLib: Building and Searching Top-Down Mass Spectral Libraries for Proteoform Identification
Source: Anal Chem. 2025 May 29;97(22):11443–53. doi: 10.1021/acs.analchem.4c06627 (PMC12163883; doi:10.1021/acs.analchem.4c06627)
Supplement: Supplementary file 1 [file ac4c06627_si_001.pdf]

Supporting Information

TopLib: Building and searching top-down mass spectral libraries for proteoform identification (Supplemental Material)

Kun Li[1], Haixu Tang[2], and Xiaowen Liu[1]*

[1]Deming Department of Medicine, Tulane University, New Orleans, Louisiana, 70112, United States

[2]Luddy School of Informatics, Computing and Engineering, Indiana University, Bloomington, Indiana, 47408, United States

*Corresponding author: xwliu@tulane.edu

Table of Contents

Supplemental Figures S-3

Figure S1. Database scheme diagram for storing MS data in TopLib....................S-3

Figure S2. The distributions of cosine similarity of same-group and different-group spectrum pairs in the SW480-SPE dataset without mass shifts and with shifts for 90% of the masses in spectra S-3

Figure S3. The distribution of the errors of matched fragment masses in the same-group spectrum pairs in the SW480-SPE dataset. S-4

Figure S4. Comparison of spectral clustering accuracy of TopCluster with the BIN representation and hierarchical clustering using three distance functions: Euclidean, Cosine, and the entropy-based distances S-4

Figure S5. Comparison of spectral clustering accuracy between hierarchical clustering and DBSCAN using TopCluster with the MASS representation. S-5

Figure S6. Comparison of spectral clustering performance between k=50 and k=100 for TopCluster using the MASS representation S-5

Figure S7. Comparison of identifications reported from spectral library searches with four parameter settings for precursor matching S-6

Figure S8. Distributions of the cosine similarity scores of the SC-SSMs and DC-SSMs of the 6,066 query spectra S-6

Figure S9. Examples of SSMs with a similarity score of 0.3 ....S-7

Figure S10. Distribution of cosine similarity scores for the 4,769 SSMs reported by searching the *E. coli* dataset against the SW480-2D-1 spectral library. S-7

Figure S11. Comparison of single representative and average representative spectra for spectral identification by searching the mass spectra in SW480-2D-2 against the SW480-2D-1 library S-8

Figure S12. PPIRs of the 5,155 spectra in the SW480-2D-1 library S-8

Figure S13. Comparison of spectral identifications reported from spectral library searches using different spectral deconvolution tools: TopFD and FLASHDeconv S-9

Figure S14. Comparison of spectral identifications reported from SW480-2D-2 using spectral library search against the SW480-2D-1 library and database search. S-9

Figure S15. Comparison of the running times for TopLib, MSPathFinder, and TopPIC in identifying spectra in the SW480-2D-2 dataset S-10

Figure S16. Comparison of the reproducibility of proteoform identifications reported by database search and spectral library search S-10

Figure S17. Comparison of database and library search results from the Triton dataset. ..S-11

Figure S18. Distribution of the number of peaks in the 5,155 non-deconvoluted MS spectra from the SW480-2D-1 library. S-11

Supplemental Tables S-12

Table S1. Parameter settings for TopFD S-12

Table S2. Parameter settings for TopPIC S-12

Table S3. Parameter settings for TopCluster in TopLib...S-13

Table S4. Comparison of different settings of k using the BIN representation on the SW480-SPE dataset with a bin size of 0.5..S-13

Table S5. Comparison of different settings of k using the DL representation on the SW480-SPE dataset S-13

Table S6. Comparison of different settings of k using the MASS representation on the SW480-SPE dataset with an error tolerance of 10 ppm S-13

Table S7. Parameter settings for FLASHDeconv S-14

Table S8. Parameter settings for MSPathFinder S-14

Supplemental Figures

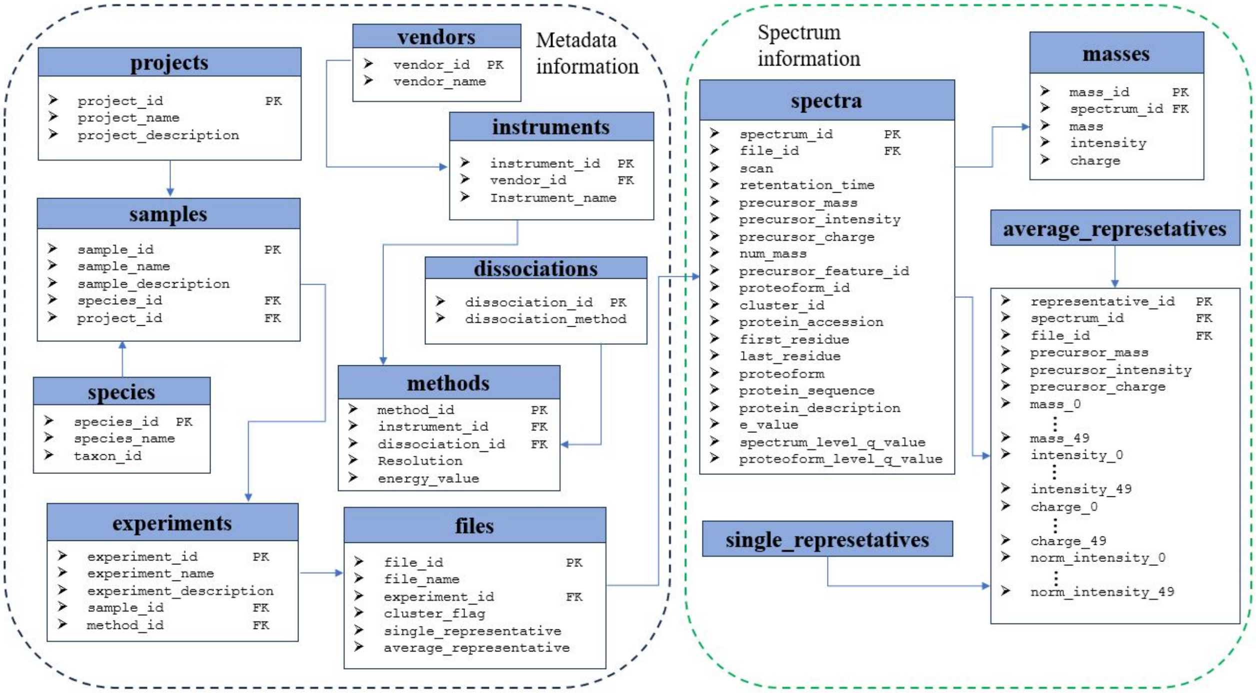

Figure S1. Database scheme diagram for storing MS data in TopLib.

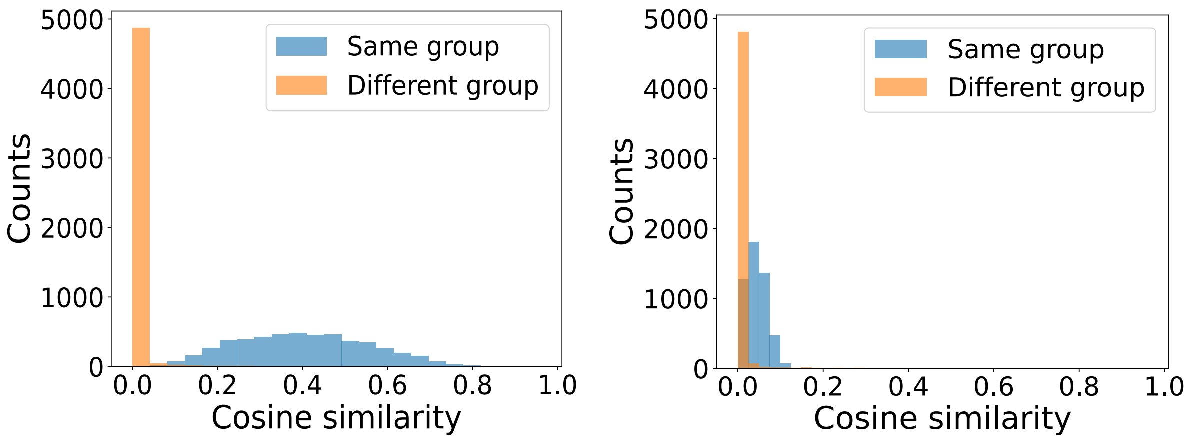

(a) (b)

Figure S2. The distributions of cosine similarity of same-group and different-group spectrum pairs in the SW480-SPE dataset without mass shifts (a) and with shifts for 90% of the masses in spectra (b).

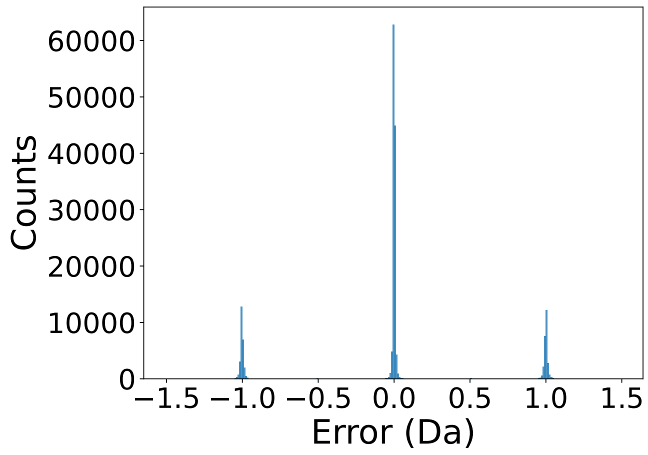

Figure S3. The distribution of the errors of matched fragment masses in the same-group spectrum pairs in the SW480-SPE dataset.

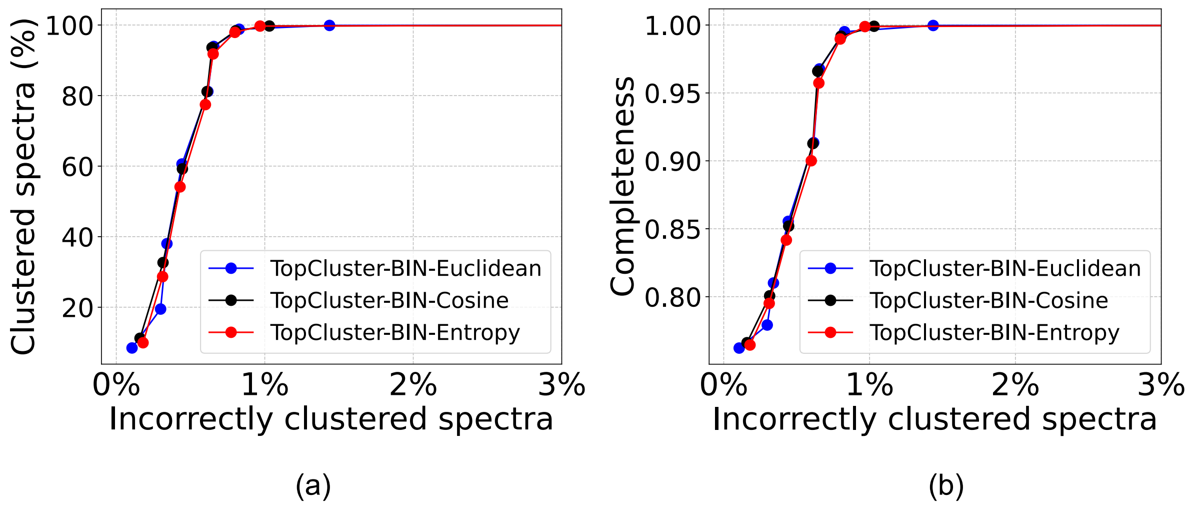

Figure S4. Comparison of spectral clustering accuracy of TopCluster with the BIN representation and hierarchical clustering using three distance functions: Euclidean, Cosine, and the entropy-based distances. (a) The ratio of incorrectly clustered spectra against the ratio of clustered spectra. (b) The ratio of incorrectly clustered spectra against clustering completeness.

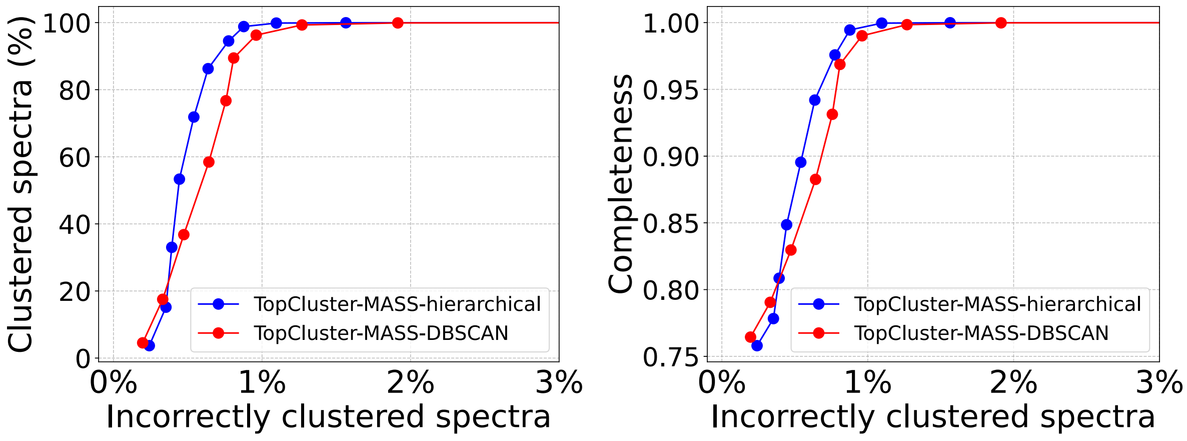

(a) (b)

Figure S5. Comparison of spectral clustering accuracy between hierarchical clustering and DBSCAN using TopCluster with the MASS representation. (a) The ratio of incorrectly clustered spectra against the ratio of clustered spectra and (b) the ratio of incorrectly clustered spectra against clustering completeness.

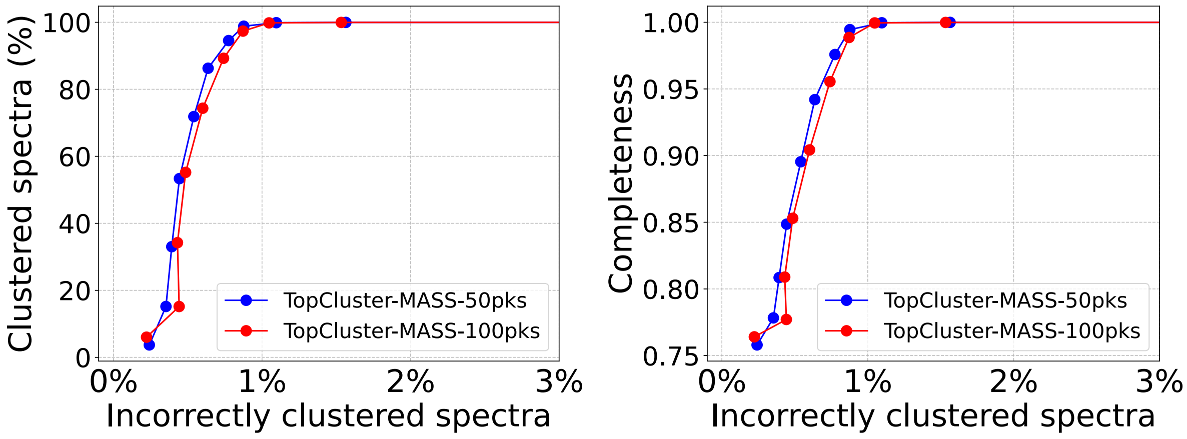

(a) (b)

Figure S6. Comparison of spectral clustering performance between k=50 and k=100 for TopCluster using the MASS representation. (a) The ratio of incorrectly clustered spectra against the ratio of clustered spectra and (b) the ratio of incorrectly clustered spectra against clustering completeness.

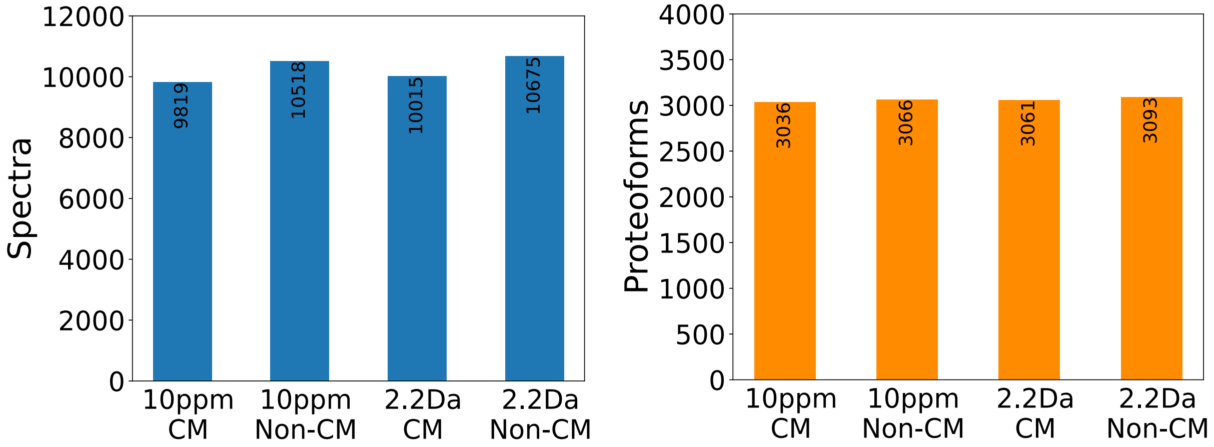

(a) (b)

Figure S7. Comparison of identifications reported from spectral library searches with four parameter settings for precursor matching. Spectra from SW480-2D-2 were searched against the SW480-2D-1 library using four parameter combinations: precursor mass error tolerance of 10 ppm or 2.2 Da, with or without precursor charge matching (CM or Non-CM). (a) Spectral identifications; (b) Proteoform identifications.

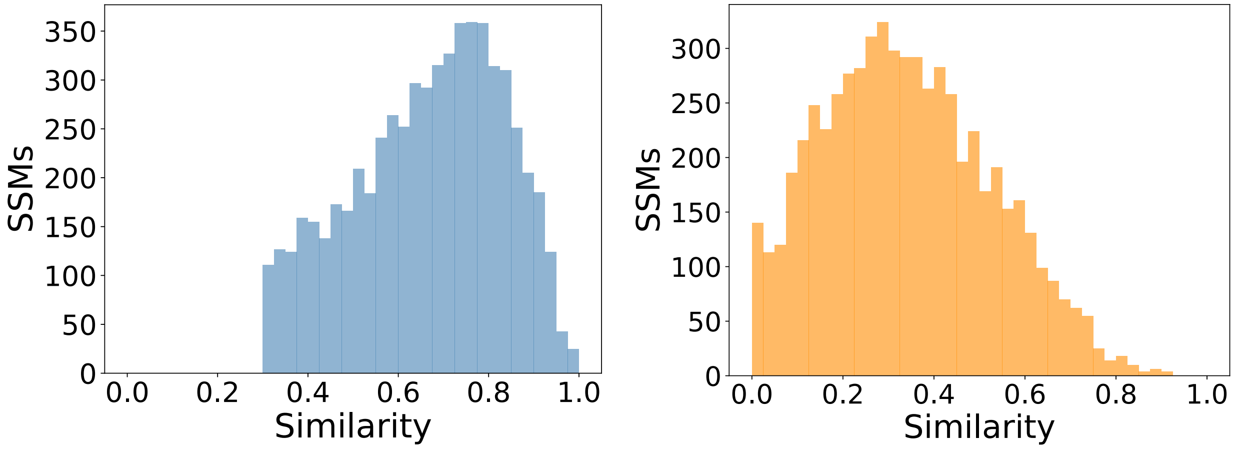

(a) (b)

Figure S8. Distributions of the cosine similarity scores of the SC-SSMs and DC-SSMs of the 6,066 query spectra. (a) SC-SSMs and (b) DC-SSMs.

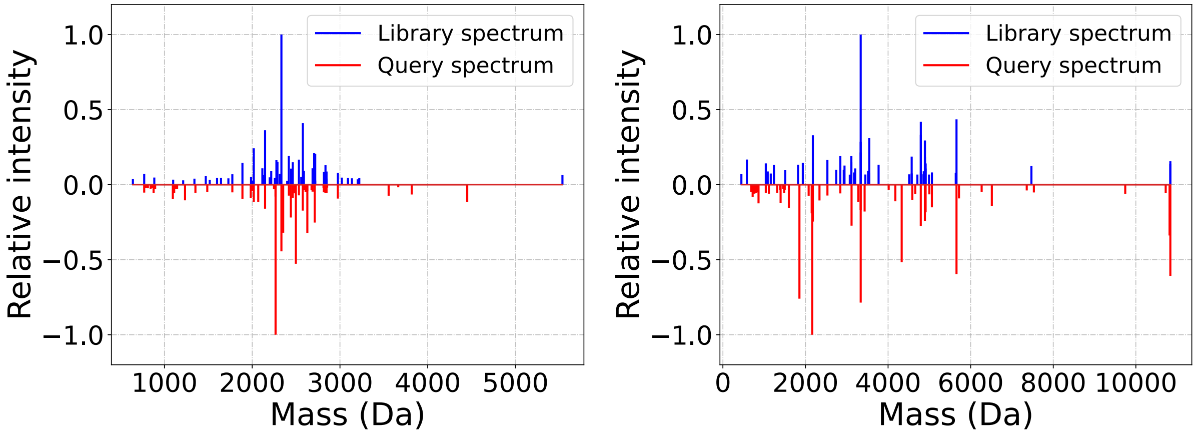

(a) (b)

Figure S9. Examples of SSMs with a similarity score of 0.3. (a) An SSM for a query spectrum with a precursor mass of 5553.20 Da. (b) An SSM for a query spectrum with a precursor mass of 10,835.88 Da.

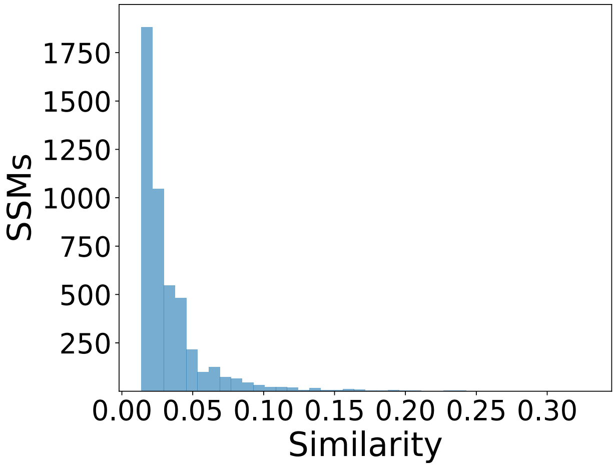

Figure S10. Distribution of cosine similarity scores for the 4,769 SSMs reported by searching the *E. coli* dataset against the SW480-2D-1 spectral library.

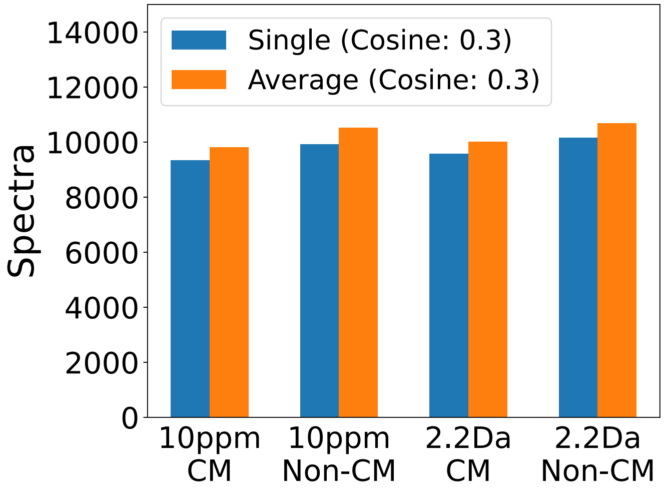

Figure S11. Comparison of single representative and average representative spectra for spectral identification by searching the mass spectra in SW480-2D-2 against the SW480-2D-1 library. The cosine similarity cutoff is set to 0.3 (Cosine: 0.3), and four different parameter combinations are used: a precursor mass error tolerance of 10 ppm or 2.2 Da, and with or without precursor charge matching (CM or Non-CM).

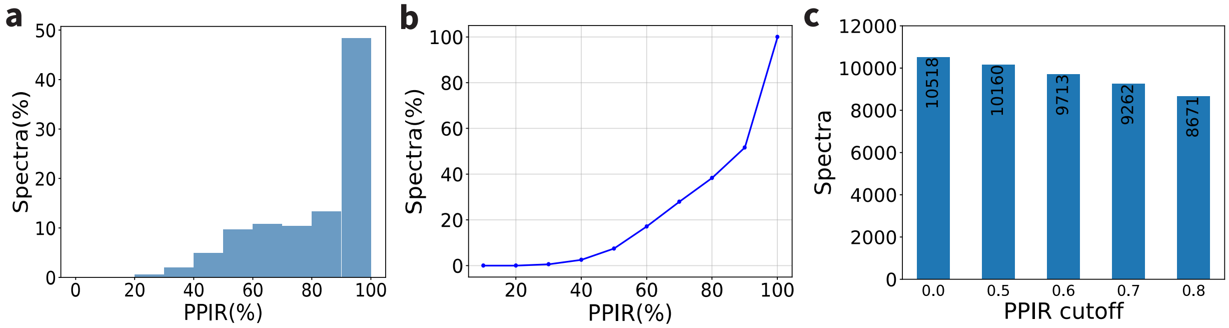

Figure S12. PPIRs of the 5,155 spectra in the SW480-2D-1 library. (a) Histogram of the PPIRs. (b) Cumulative distribution of the PPIRs. (c) Comparison of spectral identifications using spectral libraries constructed with different PPIR cutoff values. Libraries were generated from the SW480-2D-1 dataset using various PPIR cutoffs, and spectra from the SW480-2D-2 dataset were searched against these libraries using default parameter settings.

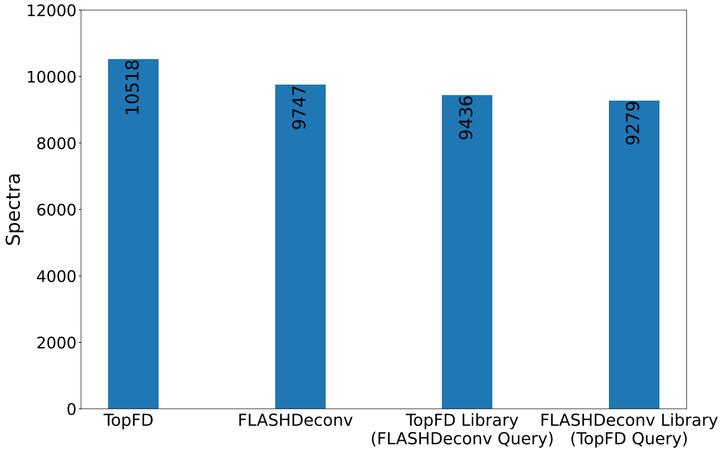

Figure S13. Comparison of spectral identifications reported from spectral library searches using different spectral deconvolution tools: TopFD and FLASHDeconv. Spectral libraries were constructed from the SW480-2D-1 dataset using TopLib, and query spectra from the SW480-2D-2 dataset were searched against these libraries. Four combinations of spectral deconvolution methods were evaluated: (1) TopFD for both library and query spectral deconvolution, (2) FLASHDeconv for both library and query spectral deconvolution, (3) TopFD for library spectral deconvolution and FLASHDeconv for query spectral deconvolution, and (4) FLASHDeconv for library spectral deconvolution and TopFD for query spectral deconvolution.

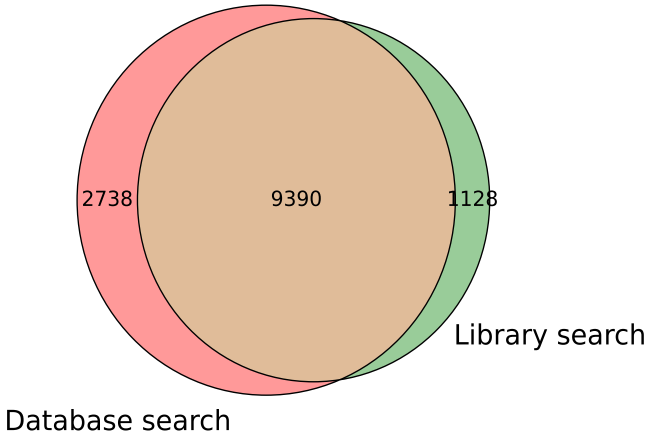

Figure S14. Comparison of spectral identifications reported from SW480-2D-2 using spectral library search against the SW480-2D-1 library and database search.

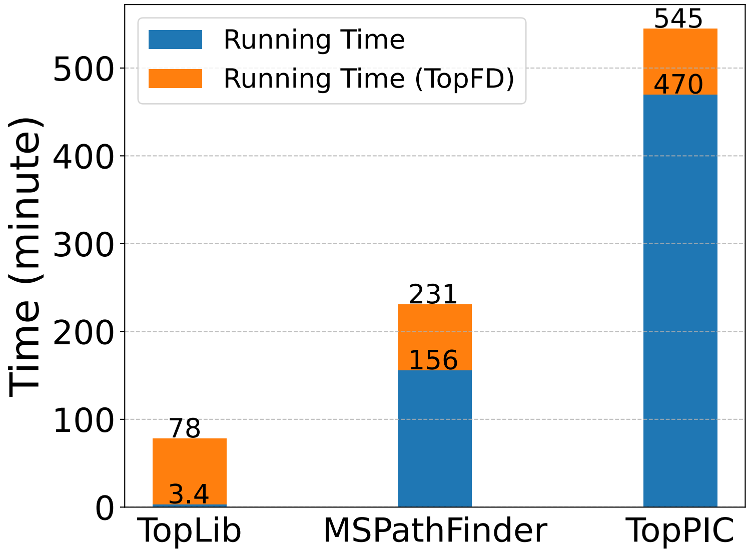

Figure S15. Comparison of the running times (blue) for TopLib, MSPathFinder, and TopPIC in identifying spectra in the SW480-2D-2 dataset. TopLib searched the spectra against the SW480-2D-1 spectral library using a single CPU thread, while MSPathFinder and TopPIC searched the spectra against the UniProt human proteome database (version July 19, 2024; 20,590 entries) using 16 CPU threads on a computer with an 11th Gen Intel Core i9-11900K 3.5 GHz CPU and 64 GB of memory. Running times for the three tools when coupled with TopFD (orange) are also shown.

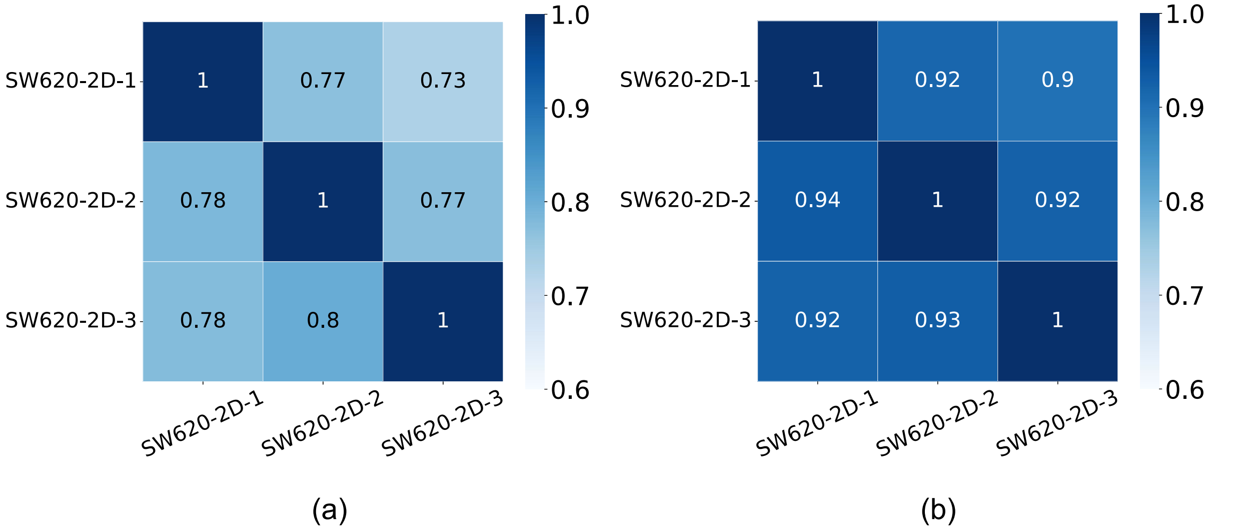

Figure S16. Comparison of the reproducibility of proteoform identifications reported by database search and spectral library search. (a) Reproducibility of proteoform identifications for the SW620-2D triplicates using database search. (b) Reproducibility of proteoform identifications using spectral library built from SW480-2D-1 for the SW620-2D triplicates.

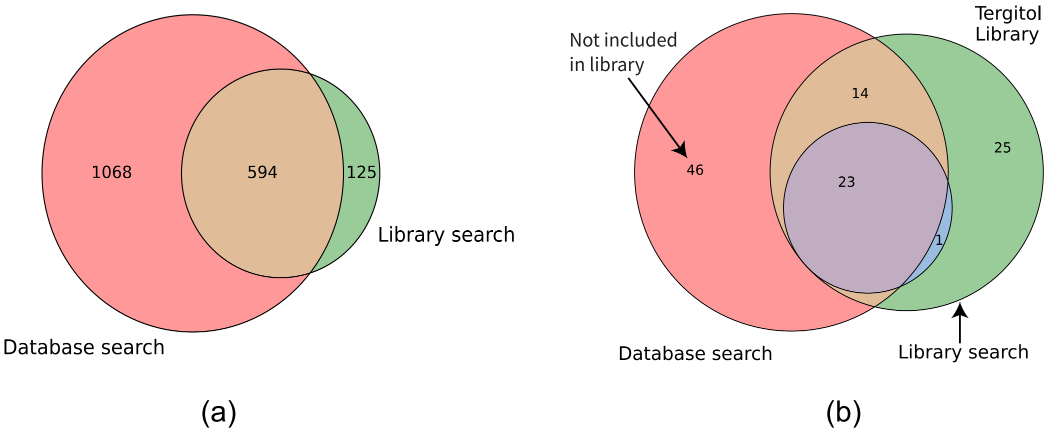

Figure S17. Comparison of database and library search results from the Triton dataset. (a) Comparison of spectral identifications reported from the Triton dataset by database search and spectral library search. (b) Comparison of proteoforms in the Tergitol spectral library, proteoform identifications reported from the Triton dataset by database search, and those by spectral library search.

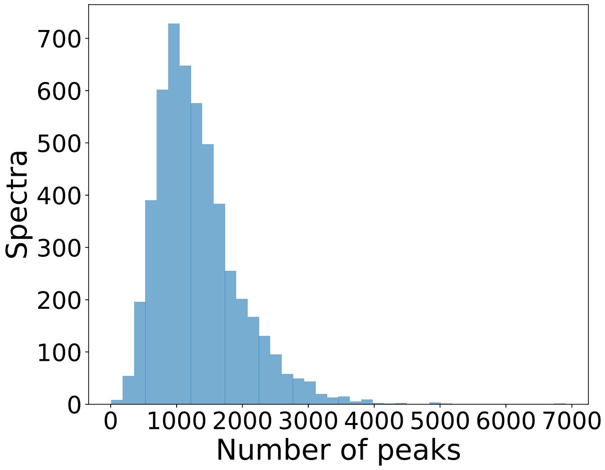

Figure S18. Distribution of the number of peaks in the 5,155 non-deconvoluted MS spectra from the SW480-2D-1 library.

Supplemental Tables

Table S1. Parameter settings for TopFD

**Parameter**	**Value**
Spectral data type	Centroid
Maximum charge	30
Maximum monoisotopic mass	70000 Dalton
Peak error tolerance	0.02 m/z
M1 signal/noise ratio	3
MS/MS signal noise ratio	1
Precursor window	3 m/z
Do final filtering	Yes

Table S2. Parameter settings for TopPIC

**Parameter**	**Value**
Proteome database	UniProt human proteome database (UP000005640_9606, 20,590 entries, version July 19, 2024)
Fragmentation method	File
Search type	Target+Decoy
N-terminal forms of proteins	NONE, M_ACETYLATION, NME, NME_ACETYLATION
Use TopFD features	Yes
Fixed modifications	No
Maximum number of variable modifications	0
Variable modifications	None
Spectrum level cutoff type for filtering PrSMs	FDR
The cutoff value for filtering PrSMs	0.01
Spectrum level cutoff type for filtering proteoforms	FDR
The cutoff value for filtering proteoforms	0.01
Error tolerance for precursor and fragment masses	10 ppm
Error tolerance for identifying PrSM clusters	10 ppm for building spectral library; 2.2 Da for estimating error rates of spectral library search.
Maximum number of unexpected mass shifts	1
Minimum value of the mass shift	-500
Maximum value of the mass shift	500
E-values computation	Generating function
Use TopFD feature file	True

Table S3. Parameter settings for TopCluster in TopLib

Function	Parameters	Settings
Precursor mass-based clustering	Clustering method	Hierarchical clustering
	Distance function	Manhattan distance
	Linkage method	Complete
	Distance cutoff	2.2 Da
MS/MS spectral clustering	Clustering method	Hierarchical clustering
	Distance function	Cosine distance
	Linkage method	Average
	Distance cutoff value	0.7

Table S4. Comparison of different settings of k using the BIN representation on the SW480-SPE dataset with a bin size of 0.5

Bin=0.5	*k*=25	*k*=50	*k*=75	*k*=100	*k*=150	*k*=200
Euclidean	0.85414	**0.87251**	0.85336	0.83435	0.80603	0.79586
Cosine	0.85500	**0.87244**	0.85335	0.83435	0.80603	0.79585
Entropy	0.85125	**0.87107**	0.86367	0.85390	0.83858	0.83327

Table S5. Comparison of different settings of k using the DL representation on the SW480-SPE dataset

	k=25	*k*=50	*k*=75	*k*=100	*k*=150	*k*=200
Euclidean	0.59197	**0.59860**	0.59215	0.58266	0.57427	0.57218
Cosine	0.59795	**0.61585**	0.61125	0.60834	0.60589	0.60646

Table S6. Comparison of different settings of k using the MASS representation on the SW480-SPE dataset with an error tolerance of 10 ppm

	k=25	*k*=50	*k*=75	*k*=100	*k*=150	*k*=200
Euclidean	0.87385	0.92455	0.92871	**0.93154**	0.93059	0.92920
Cosine	0.87408	0.92306	0.93020	**0.93178**	0.93016	0.92956
Entropy	0.87529	0.92571	0.93391	**0.93618**	0.93533	0.93507

Table S7. Parameter settings for FLASHDeconv

Parameter	Value
Minimum precursor signal noise ratio	1
mzML mass charge	0
Preceding MS1 count	3
Maximum MS level	2
Merging method	0
Tolerance	[10.0,10.0]
Output mass range	Min 100, Max 70000
Charge range	Min 1, Max 30
m/z range	Min -1, Max -1
RT range	Min -1, Max -1
Minimum isotope cosine	[0.8,0.8]
Minimum intensity	100
Mass error (ppm)	10
Mass error (Da)	-0.1
Quant method	Area
Minimum sample rate	0.2
Minimum trace length	1
Maximum trace length	-1
Minimum isotope cosine	-1

Table S8. Parameter settings for MSPathFinder

Parameter	Value
Internal cleavage mode	1
Threads	16
Database search mode	Target + decoy
Precursor tolerance (ppm)	10
Fragment ion tolerance (ppm)	10
Minimum sequence length	21
Maximum sequence length	300
Minimum precursor ion charge	1
Maximum precursor ion charge	30
Minimum fragment ion charge	1
Maximum fragment ion charge	30
Minimum sequence mass (Da)	3000
Maximum sequence mass (Da)	70000
Activation method	HCD
Static modifications	Carbamidomethyl (C), any position
Dynamic modifications	None
